# Disease Burden and Patient-Reported Outcomes Among Ulcerative Colitis Patients According to Therapy at Enrollment Into CorEvitas’ Inflammatory Bowel Disease Registry

**DOI:** 10.1093/crocol/otac007

**Published:** 2022-03-08

**Authors:** Raymond K Cross, April N Naegeli, Ryan W Harrison, Page C Moore, Rachel H Mackey, Margaux M Crabtree, Celeste A Lemay, Vipin Arora, Nathan Morris, Angelina Sontag, Cem Kayhan, Joshua R Korzenik

**Affiliations:** Division of Gastroenterology and Hepatology, University of Maryland School of Medicine, Baltimore, Maryland, USA; Eli Lilly and Company, Indianapolis, Indiana, USA; CorEvitas, LLC, Waltham, Massachusetts, USA; CorEvitas, LLC, Waltham, Massachusetts, USA; CorEvitas, LLC, Waltham, Massachusetts, USA; Department of Epidemiology, University of Pittsburg Graduate School of Public Health, Pittsburgh, Pennsylvania, USA; CorEvitas, LLC, Waltham, Massachusetts, USA; CorEvitas, LLC, Waltham, Massachusetts, USA; Eli Lilly and Company, Indianapolis, Indiana, USA; Eli Lilly and Company, Indianapolis, Indiana, USA; Eli Lilly and Company, Indianapolis, Indiana, USA; Eli Lilly and Company, Indianapolis, Indiana, USA; Division of Gastroenterology, Hepatology, and Endoscopy, Brigham and Women’s Hospital, Boston, Massachusetts, USA

**Keywords:** ulcerative colitis, registry, biologics, patient-reported outcomes, real-world data

## Abstract

**Background:**

To evaluate disease burden and patient-reported outcomes (PROs) of ulcerative colitis (UC) patients at enrollment into CorEvitas’ Inflammatory Bowel Disease Registry by therapy class.

**Methods:**

Between May 3, 2017 and September 3, 2019, 773 UC registry patients were categorized by therapy class at enrollment: patients on 5-aminosalicylic acids (5-ASAs) only (*n* = 290), and patients on biologics/Janus kinase inhibitors (JAKi) alone or in combination with 5-ASAs or immunosuppressant therapies (BIO/JAKi) (*n* = 315). To quantify between group differences, the mean/proportional differences and corresponding 95% CIs were calculated.

**Results:**

Among 605 UC patients at enrollment, BIO/JAKi patients were younger (44.1 vs. 50.9 years) more were female (58.0% vs. 49.7%), had lower remission (45.4% vs. 60.0%), had more moderate/severe disease (16.5% vs. 7.1%), experienced less proctitis (10.5% vs. 22.1%), but more pancolitis (54.6% vs. 34.1%), more corticosteroid experience (70.8% vs. 44.5%), previous biologic experience (1 prior: 21.6% vs. 2.4%; 2+ prior: 12.1% vs. 0.3%), and shorter duration of current UC therapy (1.6 vs. 3.5 years) than 5-ASAs patients. BIO/JAKi patients had higher current employment than 5-ASAs patients (70.7% vs. 62.4%) and higher mean Work Productivity and Activity Impairment (WPAI) domains for absenteeism (7.3 vs. 2.8) and activity impairment (22.0 vs. 17.5).

**Conclusions:**

Among UC patients in a real-world setting, BIO/JAKi patients had less remission, more moderate-to-severe disease, and worse PROs than 5-ASAs patients. These results suggest that despite increased therapeutic options, patients with UC currently being treated with biologics or JAKi may still experience disease burden and continued unmet needs.

## Introduction

The clinical characteristics and outcomes of ulcerative colitis (UC) patients on the available treatments are not well understood. Clinical trials data are limited and fail to capture disease burden and the spectrum of clinical response completely. Residual or persistent symptoms have not been adequately explored, particularly with respect to the differences between the newer therapies, including biologics and tofacitinib, and older therapies. Patient characteristics and outcomes across different treatments can be depicted using systematically collected real-world data, thus providing the opportunity to gain insight into the disease burden in UC patients. The examination of real-world data may inform health care providers on ways to improve treatment strategies and to tailor therapy for individual patients to achieve optimal disease control.

To address the disease burden in UC patients in a real-world setting, we describe the characteristics of patients with UC enrolled in CorEvitas’ Inflammatory Bowel Disease (IBD) Registry, by therapy class (5-ASAs alone [5-ASAs] compared to biologics/Janus kinase inhibitors (JAKi) alone or in combination with immunosuppressant therapies [ISTs] or 5-ASAs [BIO/JAKi]). Variables of interest included sociodemographic characteristics, history of comorbidities, disease activity, disease characteristics, therapy history, and patient-reported outcomes (PROs).

## Materials and Methods

### Study Setting

Launched in May 2017, CorEvitas’ IBD Registry is a prospective, noninterventional, US research registry of patients who are at least 18 years of age or older with IBD under the care of a board-certified gastroenterologist. To better focus on the study of safety and effectiveness of biologic and small molecule therapies explicitly used in the treatment of UC and Crohn’s disease (CD), in January 2019, the Registry changed the protocol to only enroll patients initiating biologic/JAKi therapy. Newly enrolling patients were required to have initiated or switched to an approved biologic or JAKi to treat UC or CD within the previous 12 months.

Eligible medications for enrollment include FDA-approved biologic treatments for IBD (eg, tumor necrosis factor inhibitors (TNFi), an interleukin-12/23 inhibitor (IL-12/23i), an integrin α4β7 inhibitor, and an integrin α4 inhibitor), and JAKi. Enrollment data are collected from the patients and their treating gastroenterologist during routine clinical visits using CorEvitas registry questionnaires. As of December 31, 2019, the CorEvitas’ IBD Registry database included 1858 patients and 3384 patient visits, with a mean duration of patient follow-up of 1.0 years (median 1.0 years). Patients were recruited from 57 private (*n* = 48) and academic (*n* = 9) practice sites across 20 states in the United States, with 123 participating gastroenterologists.

### Study Population

Between May 3, 2017 and September 3, 2019, CorEvitas’ IBD Registry enrolled 773 UC patients. Patients were categorized by UC therapy reported by their physician at the enrollment visit. UC medications included 5-ASAs (mesalamine, balsalazide, sulfasalazine), ISTs (methotrexate, 6 mercaptopurine, azathioprine, tacrolimus, cyclosporine, other ISTs), or biologics/JAKi (TNFi: adalimumab and its biosimilar, certolizumab, golimumab, infliximab, and its biosimilar; IL-12/23i: ustekinumab; integrin α4β7 inhibitor: natalizumab; integrin α4 inhibitor: vedolizumab; JAKi: tofacitinib). Our study included 2 mutually exclusive cohorts based on UC therapy at enrollment: (1) patients on 5-ASAs alone (5-ASAs); and (2) patients on biologics/JAKi alone or in combination with ISTs or 5-ASAs (BIO/JAKi). Due to a small sample size, patients using none of these therapies (*n* = 132) or IST with or without 5-ASAs (*n* = 36) were excluded.

### Assessment of Patient Characteristics and Outcomes

Data were collected for sociodemographic characteristics, history of comorbidities, disease activity, disease characteristics, therapy history, and PROs. Sociodemographic characteristics included age, gender, race (White or non-White), ethnicity (Hispanic or non-Hispanic), body mass index (BMI), type of health insurance plan, and education. History of physician-reported comorbidities included: cardiovascular, autoimmune, gastrointestinal, respiratory, digestive/hepatic and neurologic diseases, cancer, infections, diabetes, osteoporosis, depression, anxiety, and other nonserious medical conditions. The modified Charlson Comorbidity Index (mCCI) was calculated as the sum comorbid conditions available in the registry.^1^ Disease activity measures included the Mayo Score (Partial) without physician global assessment and by physician’s global assessment (PGA) (remission [0–1], mild disease [2–4], moderate disease [5–6], severe disease [7–9]). Disease characteristics included location of disease (proctitis, left-sided, pancolitis), history of hospitalization and Emergency Room (ER) use for UC-related issues, and difference in time between symptoms and UC diagnosis (years). History of extraintestinal manifestations (eg, arthritis, skin manifestations, eye involvement) was also collected.

Data included the history of previous drug therapies including the number of prior biologics and JAKi, prior 5-ASAs, prior ISTs, corticosteroid use, antibiotic use, and the duration of current UC therapy. PROs to measure health status and functioning included the Patient Reported Outcomes Measurement Information System (PROMIS) and the Work Productivity and Activity Impairment (WPAI) questionnaires. We included PROMIS measures for fatigue, sleep disturbance, pain interference, depression, and anxiety. PROMIS uses a *T*-score metric in which 50 is the mean of a relevant reference population (eg, US general population) and 10 is the SD of that population. Higher scores indicate a worse outcome and more severity of the domain being measured.^2^ PROMIS scores are grouped as within normal limits (<55), mild (55 to <60), moderate (60 to <70), and severe (≥70) based on domain scores measured in the US general population.^2,3^

The WPAI measures absenteeism (work hours missed), presenteeism (impairment at work/reduced on-the-job effectiveness), work productivity loss (overall work impairment/absenteeism plus presenteeism), and activity impairment (daily activities impaired). The WPAI outcomes are scored as percentages of impairment, with higher numbers indicating more significant impairment and less productivity (ie, worse outcomes).^4^

### Statistical Analyses

Descriptive statistics characterized patients at enrollment overall and within each cohort separately to highlight differences in patient characteristics across cohorts. Categorical variables were summarized using frequency counts and percentages. Continuous variables were summarized by the number of observations, mean, and SD. To quantify between group differences, the mean or proportional differences and their corresponding 95% CIs were calculated. Compared to *P* values, CIs aide clinical interpretation by reporting a plausible range of values in the actual units of data measured along with the direction and strength of the effect.^5^ Confidence intervals for differences that do not include 0 are considered statistically noteworthy. The outcome comparisons are cross-sectional and were not adjusted for these cohort differences and should not be interpreted as a difference in response to treatment. R version 3.6.2 (The R Foundation for Statistical Computing) was used for analyses.

### Ethical Considerations

All participating IBD Registry investigators were required to obtain full board approval for conducting research involving human subjects. Sponsor approval and continuing review were obtained through a central Institutional Review Board (IRB) (IntegReview, Protocol number Corrona-IBD-600). For academic investigative sites that did not receive a waiver to use the central IRB, approval was obtained from the respective governing IRBs, and documentation of approval was submitted to the Sponsor before initiating any study procedures. All registry subjects were required to provide written informed consent before participating.

## Results

To focus on the 5-ASAs and BIO/JAKi treated patients, the current study excluded the 132 patients not on therapy and 36 patients on IST alone or combined with 5-ASAs from the 773 UC patients enrolled in CorEvitas’ IBD Registry. The final study population at enrollment (*n* = 605) included 290 5-ASAs patients and 315 BIO/JAKi patients.

Sociodemographic characteristics, history of comorbidities, disease activity, disease characteristics, and history of extraintestinal manifestations at enrollment for patients are presented in Table 1. We found BIO/JAKi patients were younger (44.1 vs. 50.9 years), were more likely female (58.0% vs. 49.7%), more likely to have private insurance (83.5% vs. 69.0%), more pancolitis (54.6% vs. 34.1%), a history of greater hospitalization for UC-related issues (51.1% vs. 29.3%), and a history of greater ER use for UC-related issues (46.0% vs. 28.0%) than 5-ASAs patients, respectively. BIO/JAKi patients had a history of less left-sided disease (35.6% vs. 42.8%) and proctitis (10.5% vs. 22.1%) than 5-ASAs patients, respectively.

**Table 1.** Sociodemographics, history of comorbidities, disease activity, disease characteristics, and history of extraintestinal manifestations at enrollment for patients with ulcerative colitis.

	Ulcerative colitis therapy groups (at beginning of enrollment visit)			
	Total	5-ASAs^a^	BIO/JAKi^a^	Difference (95% CI)^b^
Sociodemographics				
Age in years	*n* = 605	*n* = 290	*n* = 315	
Mean (SD)	47.3 (17.0)	50.9 (17.5)	44.1 (15.7)	6.8 (4.1, 9.5)
Gender—female	*n* = 604	*n* = 290	*n* = 314	
*n* (%)	326 (54.0%)	144 (49.7%)	182 (58.0%)	−8.3 (−16.2, −0.4)
White race	*n* = 605	*n* = 290	*n* = 315	
*n* (%)	520 (86.0%)	244 (84.1%)	276 (87.6%)	−3.5 (−9.0, 2.1)
Body mass index (BMI) (kg/m^2^) categorical, *n* (%)	*n* = 602	*n* = 289	*n* = 313	
Underweight (<18.5)	12 (2.0%)	5 (1.7%)	7 (2.2%)	−0.5 (−2.7, 1.7)
Normal (18.5–25)	201 (33.4%)	93 (32.2%)	108 (34.5%)	−2.3 (−9.9, 5.2)
Overweight (25–30)	207 (34.4%)	95 (32.9%)	112 (35.8%)	−2.9 (−10.5, 4.7)
Obese (≥30)	182 (30.2%)	96 (33.2%)	86 (27.5%)	5.7 (−1.6, 13.1)
Insurance—private	*n* = 605	*n* = 290	*n* = 315	
*n* (%)	463 (76.5%)	200 (69.0%)	263 (83.5%)	−14.5 (−21.2, −7.8)
Education—college graduate or higher	*n* = 602	*n* = 289	*n* = 313	
*n* (%)	313 (52.0%)	138 (47.8%)	175 (55.9%)	−8.2 (−16.1, −0.2)
History of comorbidities				
CVD^1^	*n* = 605	*n* = 290	*n* = 315	
*n* (%)	62 (10.2%)	35 (12.1%)	27 (8.6%)	3.5 (−1.4, 8.4)
Autoimmune^2^	*n* = 605	*n* = 290	*n* = 315	
*n* (%)	12 (2.0%)	4 (1.4%)	8 (2.5%)	−1.2 (−3.4, 1.0)
Gastrointestinal^3^	*n* = 605	*n* = 290	*n* = 315	
*n* (%)	9 (1.5%)	5 (1.7%)	4 (1.3%)	0.5 (−1.5, 2.4)
Respiratory^4^	*n* = 605	*n* = 290	*n* = 315	
*n* (%)	43 (7.1%)	20 (6.9%)	23 (7.3%)	−0.4 (−4.5, 3.7)
Digestive/hepatic^5^	*n* = 605	*n* = 290	*n* = 315	
*n* (%)	17 (2.8%)	7 (2.4%)	10 (3.2%)	−0.8 (−3.4, 1.9)
Cancer^6^	*n* = 605	*n* = 290	*n* = 315	
*n* (%)	35 (5.8%)	15 (5.2%)	20 (6.3%)	−1.2 (−4. 9, 2.5)
Neurologic^7^	*n* = 605	*n* = 290	*n* = 315	
*n* (%)	14 (2.3%)	7 (2.4%)	7 (2.2%)	0.2 (−2.2, 2.6)
Other^8^	*n* = 605	*n* = 290	*n* = 315	
*n* (%)	218 (36.0%)	89 (30.7%)	129 (41.0%)	−10.3 (−17.9, −2.7)
Infections^9^	*n* = 605	*n* = 290	*n* = 315	
*n* (%)	75 (12.4%)	30 (10.3%)	45 (14.3%)	−4.0 (−9.2, 1.3)
Modified Charlson Comorbidity Index^10^	*N* = 605	*n* = 290	*n* = 315	
Mean (SD)	0.2 (0.5)	0.2 (0.5)	0.2 (0.5)	0.01 (−0.08, 0.09)
Disease activity				
Mayo Score (Partial), w/o physician global assessment (0–6)	*n* = 579	*n* = 286	*n* = 293	
Mean (SD)	1.0 (1.3)	0.8 (1.2)	1.2 (1.4)	−0.3 (−0.6, −0.1)
Physician’s global assessment, *n* (%)	*n* = 578	*n* = 285	*n* = 293	
Normal or inactive disease (0–1)	211 (36.5%)	100 (35.1%)	111 (37.9%)	−2.8 (−10.6, 5.1)
Mild disease (2–4)	240 (41.5%)	151 (53.0%)	89 (30.4%)	22.6 (14.8, 30.4)
Moderate disease (5–6)	101 (17.5%)	33 (11.6%)	68 (23.2%)	−11.6 (−17.7, −5.5)
Severe disease (7–9)	26 (4.5%)	1 (0.4%)	25 (8.5%)	−8.2 (−11. 5, −4.9)
Disease characteristics				
Proctitis	*n* = 605	*n* = 290	*n* = 315	
*n* (%)	97 (16.0%)	64 (22.1%)	33 (10.5%)	11.6 (5.7, 17.4)
Left-sided disease	*n* = 605	*n* = 290	*n* = 315	
*n* (%)	236 (39.01%)	124 (42.8%)	112 (35.6%)	7.2 (−0.57, 14.97)
Pancolitis	*n* = 605	*n* = 290	*n* = 315	
*n* (%)	271 (44.8%)	99 (34.1%)	172 (54.6%)	−20.5 (−28.2, −12.7)
History of hospitalization for UC-related issues	*n* = 603	*n* = 290	*n* = 313	
*n* (%)	245 (40.6%)	85 (29.3%)	160 (51.1%)	−21.8 (−29.4, −14.2)
History of ER use for UC-related issues	*n* = 602	*n* = 289	*n* = 313	
*n* (%)	225 (37.4%)	81 (28.0%)	144 (46.0%)	−18.0 (−25.6, −10.4)
Difference in time between symptoms and UC diagnosis (years)	*n* = 593	*n* = 287	*n* = 306	
Mean (SD)	0.9 (3.2)	0.6 (1.8)	1.2 (4.0)	−0.5 (−1.0, −0.1)
History of extraintestinalmanifestations				
Arthritis	*n* = 605	*n* = 290	*n* = 315	
*n* (%)	59 (9.8%)	15 (5.2%)	44 (14.0%)	−8.8 (−13.4, −4.2)
Skin manifestations	*n* = 605	*n* = 290	*n* = 315	
*n* (%)	7 (1.2%)	0 (0.0%)	7 (2.2%)	−2.2 (−3.9, −0.6)
Eye involvement	*n* = 605	*n* = 290	*n* = 315	
*n* (%)	2 (0.3%)	0 (0.0%)	2 (0.6%)	−0.6 (−1.5, 0.2)

Abbreviations: 5-ASAs, 5-aminosalicylic acids; ER, Emergency Room; ISTs, immunosuppressant therapies; JAKi, Janus kinase inhibitors; 5-ASAs alone (5-ASAs); biologics/JAKi alone or in combination with ISTs or 5-ASAs (BIO/JAKi). 1 includes cardiac revascularization procedure, ventricular arrhythmia, cardiac arrest, myocardial infarction, acute coronary syndrome, unstable angina, coronary artery disease, congestive heart failure, and cerebrovascular disease (stroke, transient ischaemic attack, peripheral vascular disease, peripheral arterial disease); 2 includes alopecia areata, alopecia totalis, autoimmune hepatitis, systemic lupus erythematosus, psoriasis, sarcoidosis, Sjogren’s syndrome, and vasculitis; 3 includes peptic ulcer, gastrointestinal perforation, and small bowel obstruction; 4 includes asthma, interstitial lung disease/pulmonary fibrosis and chronic obstructive pulmonary disease (COPD); 5 includes primary sclerosing cholangitis, cholelithiasis, and other hepatic event (serious or requiring biopsy); 6 includes colonic dysplasia, colon cancer, lymphoma, lung cancer, breast cancer, skin cancer (basal cell), skin cancer (melanoma), premalignancy, and other cancer; 7 includes progressive multifocal leukoencephalopathy, demyelinating disease, amyotrophic lateral sclerosis, multiple sclerosis, fibromyalgia, and other neurological disorder (serious); 8 includes diabetes, osteoporosis, depression, anxiety, and other nonserious medical conditions; 9 includes bronchitis, *C. difficile* colitis, *Candida*, diverticulitis, gastroenteritis, herpes zoster, joint/bursa, meningitis/encephalitis, pneumonia, sepsis, sinusitis, upper respiratory infection (URI), urinary tract infection (UTI), tuberculosis (TB), and other infection; 10 The modified Charlson Comorbidity Index (mCCI) was calculated as the sum of prior (history of) physician-reported comorbid conditions in the CorEvitas’ IBD Registry, including myocardial infarction, congestive heart failure, peripheral vascular disease, cerebrovascular disease, COPD, peptic ulcer disease, diabetes mellitus, lymphoma, solid-tumor cancer (excluding nonmelanoma of the skin), mild liver disease (hepatic events), and moderate/severe liver disease (primary sclerosing cholangitis, autoimmune hepatitis). Conditions that were not captured and were excluded from mCCI score include connective tissue disease, dementia, kidney disease, hemiplegia, and acquired immunodeficiency syndrome.

Concomitant (eg, currently taking) use of steroids or antibiotics at the time of enrollment is allowed in the UC therapy groups.

Unadjusted mean difference (95% CI) between groups for continuous variables and difference in percentage points (95% CI) between groups for categorical variables.

**Figure 1.**
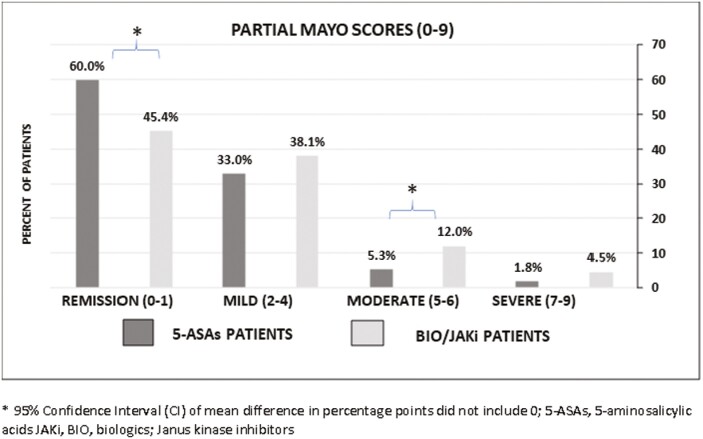
Disease severity at enrollment for patients with UC. *95% CI of mean difference in percentage points did not include 0. Abbreviations: 5-ASAs, 5-aminosalicylic acids; BIO/JAKi, biologics, Janus kinase inhibitors; UC, ulcerative colitis.

Figure 1 presents disease severity at enrollment for patients based on the Partial Mayo Score. BIO/JAKi patients had more moderate (Partial Mayo Score [5–6], 12.0% vs. 5.3%) and severe (Partial Mayo Score [7–9], 4.5% vs. 1.8%) disease and less remission (Partial Mayo Score [0–1], 45.4% vs. 60.0%) than 5-ASAs patients, respectively.

The history of prior UC therapies at enrollment for patients is presented in Table 2. Compared to 5-ASAs patients, BIO/JAKi patients had a shorter mean duration of current UC therapy (1.6 vs. 3.5 years), had more corticosteroid experience (70.8% vs. 44.5%), and previous biologic experience (1 prior: 21.6% vs. 2.4%; 2+ prior: 12.1% vs. 0.3%), respectively.

**Table 2.** History of prior ulcerative colitis therapies at enrollment for patients with ulcerative colitis.

	Ulcerative colitis therapy groups (at beginning of enrollment visit)			
	Total	5-ASAs^a^	BIO/JAKi^a^	Difference (95% CI)^b^
Prior UC therapies^c^				
Biologic experienced	*n* = 605	*n* = 290	*n* = 315	
*n* (%)	323 (53.39%)	8 (2.76%)	315 (100.00%)	−97.2 (−99.1, −95.4)
Number of prior biologics^d^, *n* (%)	*n* = 605	*n* = 290	*n* = 315	
0	491 (81.2%)	282 (97.2%)	209 (66.3%)	30.9 (25.3, 36.4)
1	75 (12.4%)	7 (2.4%)	68 (21.6%)	−19.1 (−24.1, −14.3)
2+	39 (6.4%)	1 (0.3%)	38 (12.1%)	−11.7 (−15.4, −8.1)
5-ASAs experienced	*n* = 605	*n* = 290	*n* = 315	
*n* (%)	517 (85.5%)	290 (100.0%)	227 (72.1%)	27.9 (23.0, 32.9)
ISTs experienced	*n* = 605	*n* = 290	*n* = 315	
*n* (%)	121 (20.0%)	11 (3.8%)	110 (34.9%)	−31.1 (−36.8, −25.4)
Corticosteroid experienced	*n* = 605	*n* = 290	*n* = 315	
*n* (%)	352 (58.2%)	129 (44.5%)	223 (70.8%)	−26.3 (−33.9, −18.7)
Antibiotic experienced	*n* = 605	*n* = 290	*n* = 315	
*n* (%)	117 (19.3%)	44 (15.2%)	73 (23.2%)	−8.0 (−14.2, −1.8)
Duration of current UC therapy (years)	*n* = 604	*n* = 289	*n* = 315	
Mean (SD)	2.5 (3.7)	3.5 (4.5)	1.6 (2.3)	1.9 (1.3, 2.5)

Abbreviations: 5-ASAs, 5-aminosalicylic acids; IST, immunosuppressant therapies; JAKi, Janus kinase inhibitors; 5-ASAs alone (5-ASAs); biologics/JAKi alone or in combination with ISTs or 5-ASAs (BIO/JAKi).

Concomitant (eg, currently taking) use of steroids or antibiotics at the time of enrollment is allowed in the UC therapy groups.

Unadjusted mean difference (95% CI) between groups for continuous variables and difference in percentage points (95% CI) between groups for categorical variables.

UC therapies included: 5-ASAs (mesalamine, balsalazide, sulfasalazine), ISTs (methotrexate, 6 mercaptopurine, azathioprine, tacrolimus, cyclosporine, other ISTs), or biologics/JAKi (tumor necrosis factor inhibitors [TNFi]: adalimumab and its biosimilar, certolizumab, golimumab, infliximab, and its biosimilar; interleukin-12/23 inhibitor [IL-12/23i]: ustekinumab; integrin α4β7 inhibitor: natalizumab; integrin α4 inhibitor: vedolizumab; JAKi: tofacitinib).

Prior to current treatment.

**Figure 2.**
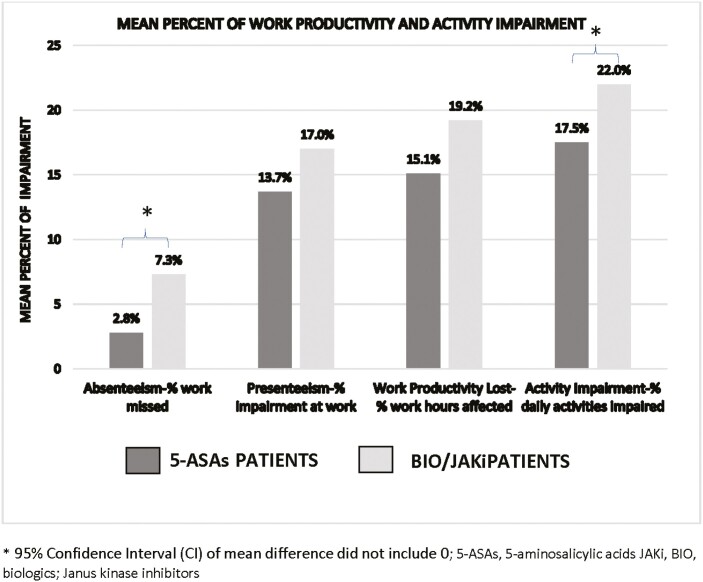
Work Productivity and Activity Impairment (WPAI) Questionnaire mean impairment scores at enrollment for patients with UC. *95% CI of mean difference did not include 0. Abbreviations: 5-ASAs, 5-aminosalicylic acids; BIO/JAKi, biologics, Janus kinase inhibitors; UC, ulcerative colitis.

Table 3 presents PROMIS scores and WPAI domains at enrollment. For both BIO/JAKi and 5-ASAs patients, the median PROMIS scores were within normal limits (<55) based on population norms. When evaluating the amount of impairment at work and impact on daily activities due to UC, BIO/JAKi patients had higher mean impairment score for absenteeism (7.3% vs. 2.8%) and activity impairment (22.0% vs. 17.5%) than 5-ASAs patients, respectively (Figure 2).

**Table 3.** History of Patient Reported Outcome Measurement Information System (PROMIS) scores and Work Productivity Activity and Impairment (WPAI) domains at enrollment for patients with ulcerative colitis.

	Ulcerative colitis therapy groups (at beginning of enrollment visit)			
	Total	5-ASAs^a^	BIO/JAKi^a^	Difference (95% CI)^b^
PROMIS^c^				
Fatigue	*n* = 595	*n* = 284	*n* = 311	
Mean (SD)	50.1 (11.1)	48.9 (10.7)	51.3 (11.4)	−2.4 (−4.2, −0.6)
Median (Q1, Q3)	49.4 (42.0, 57.5)	49.4 (42.0, 56.3)	50.9 (44.2, 60.0)	
Sleep disturbance	*n* = 601	*n* = 289	*n* = 312	
Mean (SD)	50.1 (8.3)	49.3 (7.8)	50.8 (8.6)	−1.6 (−2.9, −−0.3)
Median (Q1, Q3)	50.5 (43.8, 54.3)	50.5 (43.8, 54.3)	50.5 (46.2, 56.1)	
Pain interference	*n* = 603	*n* = 289	*n* = 314	
Mean (SD)	49.3 (9.4)	49.4 (9.3)	49.3 (9.5)	0.0 (−1.5, 1.5)
Median (Q1, Q3)	41.6 (41.6, 55.6)	41.6 (41.6, 55.6)	41.6 (41.6, 55.6)	
Depression	*n* = 602	*n* = 289	*n* = 313	
Mean (SD)	46.6 (8.0)	46.0 (7.7)	47.1 (8.3)^c^	−−1.1 (-2.4, 0.1)
Median (Q1, Q3)	41.0 (41.0, 51.8)	41.0 (41.0, 51.8)	41.0 (41.0, 51.8)	
Anxiety	*n* = 601	*n* = 289	*n* = 312	
Mean (SD)	50.4 (9.7)	49.6 (9.4)	51.2 (9.9)	−1.6 (−3.2, −0.1)
Median (Q1, Q3)	51.2 (40.3, 57.7)	48.0 (40.3, 57.7)	51.2 (40.3, 59.5)	
WPAI				
Current employment	*n* = 604	*n* = 290	*n* = 314	
*n* (%)	403 (66.7%)	181 (62.4%)	222 (70.7%)	−8.3 (−15.8, −0.8)
Absenteeism	*n* = 368	*n* = 162	*n* = 206	
Mean (SD)	5.3 (18.2)	2.8 (12.0)	7.3 (21.8)	−4.5 (−8.0, −1.0)
Presenteeism	*n* = 399	*n* = 180	*n* = 219	
Mean (SD)	15.5 (24.4)	13.7 (22.6)	17.0 (25.6)	−3.3 (−8.1, 1.4)
Work productivity loss	*n* = 367	*n* = 162	*n* = 205	
Mean (SD)	17.4 (26.3)	15.1 (23.6)	19.2 (28.2)	−4.0 (−9.4, 1.3)
Activity impairment	*n* = 603	*n* = 290	*n* = 313	
Mean (SD)	19.8 (27.0)	17.5 (25.6)	22.0 (28.0)	−4.6 (−8.9, −0.3)

Concomitant use of steroids or antibiotics at the time of enrollment is allowed in the UC therapy groups.

Unadjusted mean difference (95% CI) between groups for continuous variables and difference in percentage points (95% CI) between groups for categorical variables.

PROMIS scores: <55 = within normal limits (WNL), 55 ≤ 60 = mild, 60 ≤ 70 = moderate, and ≥70 = severe based on the US general population.

## Discussion

Because real-world systematically collected data provide a clearer picture of the burden of disease in UC, the objective of this study was to describe characteristics of patients with UC, including demographics, history of comorbidities, disease activity, disease characteristics, therapy history, and PROs, by therapy, in a US population-based IBD Registry. Our study found that those UC patients who received advanced therapies (BIO/JAKi) had a history of more severe disease, and worse clinical outcomes including hospitalization and ER use for UC-related issues, work absenteeism, and impaired daily activities compared to patients receiving 5-ASAs.

In the clinical setting, patients with mild-to-moderate UC disease severity are treated with 5-ASAs, and biologics/JAKi are used when 5-ASAs do not adequately control disease. Although biologic therapies have been available for IBD for over 20 years, patient outcomes have not generally changed over this time. According to the American Gastroenterological Association (AGA) recommendations for managing UC patients with moderate-to-severe disease activity and/or who are at high risk of colectomy, clinicians should implement biologic agents with or without an immunomodulator early rather than gradual step-up therapy after failure of 5-ASAs.^6^ Given the prior therapy history in the BIO/JAKi cohort, it is likely that patients in this group had treatment refractory disease. The differences between BIO/JAKi and 5-ASAs are likely due to differences in disease severity between groups and not necessarily the effects of treatments.

Our findings correlate with the two most recent real-world studies addressing treatment patterns^7^ and the association between disease activity and quality of life^8^ among moderate-to-severe UC patients in the United States and Europe. In the first study, Armuzzi et al found many patients with moderate-to-severe IBD initiated advanced treatments early in their disease course and that combining ISTs and biologics was common, as conventional therapies were not considered an adequate initial treatment.^7^ In the second study, the same researchers found that patients with moderate-to-severe UC treated with biologics still had significant impairment in health-related quality of life, including work and daily activities.^8^ To achieve the best possible long-term outcomes, control of clinical symptoms and achieving mucosal healing is vital. Thus, defining unmet needs in UC patients is paramount in increasing the awareness of the problems associated with current therapeutic management.^9^

Due to an increase in the number of pharmacologic agents available to treat moderate-to-severe UC over the last 5 years, more exploration is needed to evaluate some critical knowledge gaps in treating UC patients. These knowledge gaps include: identifying biomarkers predictive of response to individual therapies to facilitate the optimal choice of treatments; developing clinical prediction models to help identify patients who have low vs. high probability of response to treatment; and creating novel combinations of available therapies in patients with moderate-to-severe UC.^10^ To further tailor treatment to individual patients, clinicians will need to determine which drugs to use and which molecular pathway to target. Identifying subpopulations of patients who respond to specific medications based on an increased understanding of pharmacogenomics, biomarkers, and clinical characteristics is vital.^11^ Implementing these suggestions while characterizing treatment patterns and sequencing in patients with UC will help researchers identify new and more efficacious treatments.

This study’s limitations include the cross-sectional analysis of observational data that did not evaluate improvement over time, specifically about patients who started biologics or advanced therapeutics. Comparisons were made to highlight the differences in patient characteristics across cohorts and should not be construed as differences in response to treatment. As with all registry-based research, selection bias may be introduced during enrollment if certain subgroups of patients are routinely included or excluded from the registry. The patient population might not be generalizable to UC patients outside the United States. Caution is warranted when interpreting these results as this was an exploratory analysis and was not adjusted for potential confounding factors. However, these unadjusted results are a preliminary step to provide data to develop future research which would include adjusted models. We believe the fact that patients with higher disease severity are more likely to be on biologics is intuitive.

In contrast to these limitations, we do not believe selection bias occurred in the CorEvitas Registry and thus did not influence our study. CorEvitas observational data consist of community practice and referral tertiary care IBD centers and includes high and low acuity patients. Our study involved real-world systematically collected data that provide a more realistic view of the burden of disease in UC patients and is more reflective of the UC population than those in clinical trials. Moreover, our results further add to the evidence that there continue to be unmet treatment needs in patients with moderate-to-severe UC.^7,9^ As with the most recent real-world studies in UC patients in the United States and Europe,^8^ many of our patients initiated advanced biologic therapies and experienced a low health-related quality of life.

## Conclusions

At enrollment in CorEvitas’ IBD Registry among UC patients in a real-world setting, patients treated with biologics and JAKi had less remission, more moderate-to-severe disease, increased absenteeism at work, and added negative impact on daily activities than those on 5-ASAs. These findings may suggest, along with recent population-based studies in UC patients, that despite increased therapeutic options, there is a significant disease burden and continued unmet needs among patients with UC currently being treated with biologics or JAKi. More research needs to be conducted to identify new and more efficacious and safe treatments that benefit patients based on clinical assessments and PROs, how clinicians can optimize treatments, and discover better methods for sequencing UC therapies.

## Data Availability

Data are available from CorEvitas, LLC through a commercial subscription agreement and are not publicly available. No additional data are available from the authors.

## References

[1] Charlson ME , Pompei P, Ales KL, MacKenzie CR. A new method of classifying prognostic comorbidity in longitudinal studies: development and validation. J Chronic Dis. 1987;40(5):373–383. 3558716 10.1016/0021-9681(87)90171-8

[2] Health Measures. PROMIS score cut points: general guidelines for interpreting PROMIS scores have been constructed using different methods. 2020. Accessed October 23, 2020. https://www.healthmeasures.net/index.php?option=com_content&view=category&layout=blog&id=200&Itemid=1213

[3] Kappelman MD , Long MD, Martin C, et al. Evaluation of the patient-reported outcomes measurement information system in a large cohort of patients with inflammatory bowel diseases. Clin Gastroenterol Hepatol. 2014;12(8):1315–1323.e2. 24183956 10.1016/j.cgh.2013.10.019 PMC4361943

[4] Reilly MC , Bracco A, Ricci JF, Santoro J, Stevens T. The validity and accuracy of the Work Productivity and Activity Impairment questionnaire—irritable bowel syndrome version (WPAI:IBS). Aliment Pharmacol Ther. 2004;20(4):459–467. 15298641 10.1111/j.1365-2036.2004.02091.x

[5] du Prel JB , Hommel G, Rohrig B, Blettner M. Confidence interval or p-value? Part 4 of a series on evaluation of scientific publications. Dtsch Arztebl Int. 2009;106(19):335–339. 19547734 10.3238/arztebl.2009.0335 PMC2689604

[6] Feuerstein JD , Isaacs KL, Schneider Y, et al. AGA clinical practice guidelines on the management of moderate to severe ulcerative colitis. Gastroenterology. 2020;158(5):1450–1461. 31945371 10.1053/j.gastro.2020.01.006 PMC7175923

[7] Armuzzi A , DiBonaventura MD, Tarallo M, et al. Treatment patterns among patients with moderate-to-severe ulcerative colitis in the United States and Europe. PLoS One. 2020;15(1):e0227914. 31945774 10.1371/journal.pone.0227914 PMC6964980

[8] Armuzzi A , Tarallo M, Lucas J, et al. The association between disease activity and patient-reported outcomes in patients with moderate-to-severe ulcerative colitis in the United States and Europe. BMC Gastroenterol. 2020;20(1):18. doi:10.1186/s12876-020-1164-0 31964359 PMC6975026

[9] Daperno M , Armuzzi A, Danese S, et al. Unmet medical needs in the management of ulcerative colitis: results of an Italian Delphi consensus. Gastroenterol Res Pract. 2019;2019:3108025. doi:10.1155/2019/3108025 31565051 PMC6745180

[10] Singh S , Allegretti JR, Siddique SM, Terdiman JP. AGA technical review on the management of moderate to severe ulcerative colitis. Gastroenterology. 2020;158(5):1465–1496.e17. 31945351 10.1053/j.gastro.2020.01.007 PMC7117094

[11] Ungaro R , Mehandru S, Allen PB, Peyrin-Biroulet L, Colombel JF. Ulcerative colitis. Lancet. 2017;389(10080):1756–1770. 27914657 10.1016/S0140-6736(16)32126-2 PMC6487890

